# A new classification of congenital abnormalities of UPVS: sonographic appearances, screening strategy and clinical significance

**DOI:** 10.1186/s13244-021-01068-5

**Published:** 2021-09-06

**Authors:** Yue Qin, Huaxuan Wen, Meiling Liang, Dandan Luo, Qing Zeng, Yimei Liao, Mengyu Zhang, Yan Ding, Xin Wen, Ying Tan, Ying Yuan, Shengli Li

**Affiliations:** 1 Department of Ultrasound, Affiliated Shenzhen Maternity and Child Healthcare Hospital, Southern Medical University, Hongli Road No. 2004, Futian, Shenzhen, 518028 Guangdong China; 2 Department of Ultrasound, Shenzhen Maternity and Child Healthcare Hospital, The First School of Clinical Medicine, Southern Medical University, Shenzhen, 518028 Guangdong China

**Keywords:** Umbilical–portal venous system, New comprehensive classification, Ultrasonographic characteristics, Screening strategy, Postnatal management

## Abstract

The umbilical–portal venous system (UPVS) plays an important role in embryonic development, as well as a significant blood circulation system to ensure the normal blood supply of fetal heart and brain and other vital organs. Congenital anomalies of UPVS contain many subtypes with a broad spectrum of manifestations and prognoses. Furthermore, because of fetal small lumen of UPVS, the sonographic evaluation remains difficult in utero. Appreciation of normal embryology and anatomy of UPVS is essential to an understanding of sonographic characteristics of anomalies of UPVS and fetal sequential changes. Through reviewing previous references and our experience with congenital abnormalities of UPVS, a new comprehensive classification is proposed. The new classification identifies three types of congenital abnormalities of UPVS based on morphological abnormalities and shunts. The embryology and etiology, sonographic, clinical and prognostic characteristics of each subtype of the new classification are described in detail. Knowledge of congenital abnormalities of UPVS can give sonographers a clue and aid prenatal sonographic diagnosis. The purpose of this article is to help the sonographers to understand the new classification of congenital abnormalities of UPVS, master the sonographic characteristics of each subtype and prenatal ultrasonographic screening strategy, and guide subsequent appropriate counseling and management.

## Key points

- Congenital anomalies of UPVS contain many subtypes with a broad spectrum of manifestations and prognoses.
- The new classification could identify morphological abnormalities of the PV and the UV, and vascular connection abnormalities.
- When some anomalies are detected, the whole UPVS, the heart function and other anatomical structures should be examined in detail.
- The prognostic characteristics of congenital anomalies of UPVS are also different.
- To master the prognostic characteristics is very important to guide subsequent appropriate counseling and management.

## Background

Umbilical–portal venous system (UPVS) develops from pairs vitelline veins and umbilical veins, including the umbilical vein (UV), the portal vein (PV), the ductus venosus (DV), the superior mesenteric vein (SMV), the splenic vein (SpV) and the inferior mesenteric vein (IMV). Fetal UV, PV and DV are relatively easy to be detected, while prenatal evaluation of other venous vessels is rare. UPVS plays an important role in the blood circulation of the fetus. In the fetus, oxygen- and nutrient-rich blood from the placenta is delivered through the UV. The DV originates from the UV at the portal sinus (PS) and bypasses the liver to drain 20–30% oxygen-containing blood preferentially into the inferior vena cava (IVC) to the left side of the heart [1]. The main portal vein (MPV) enters the liver in the porta hepatis, posterior to the hepatic artery and the common hepatic duct. The hepatic veins as the efferent venous drainage of the liver drain into the IVC to the right side of the heart.

The prenatal sonographic assessment of fetal UPVS has developed to a large extent in recent years [1–9]. Most previous classifications focus on the shunt between UPVS and the systemic veins or arteries, but not anatomy abnormalities of UPVS. The aim of this review is to propose a new classification of congenital abnormal UPVS and prenatal ultrasonographic screening strategy, to analyze the clinical and prognostic characteristics, in order to enable better prenatal counseling and postnatal management.

### Normal anatomy of fetal UPVS

**Fig. 1:**
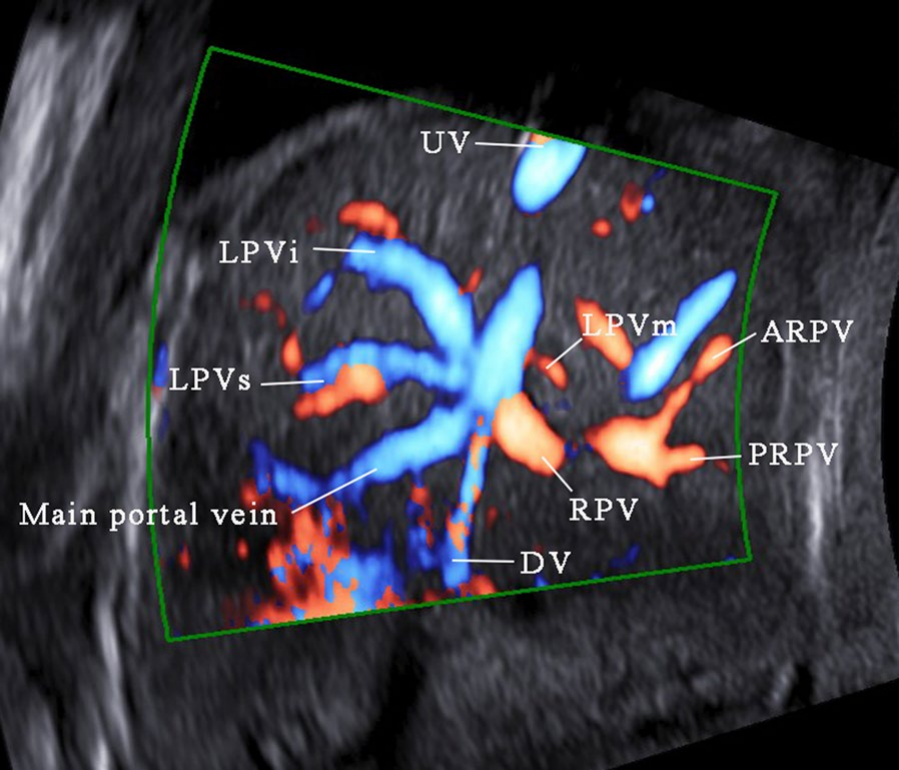
The ultrasonographic image of the normal fetal umbilical–portal venous system

The UV originates from placental villous capillary, accompanying with umbilical artery in the umbilical cord. The UV enters the abdomen at the umbilicus and then enters the liver along the ligamentum falciforme. The intrahepatic part of the UV is short, which then merges with the left portal vein (LPV). The LPV could be detected in a transverse plane of the upper abdomen presenting as an L-shaped vein, composed of the umbilical segment and the pars transversa of the LPV [2]. In the same course of the UV, the DV arises from the PS and bypasses the liver to drain blood into the IVC. The LPV bifurcates into three branches, the inferior (LPVi), middle (LPVm) and superior (LPVs). The MPV merges with the LPV and gives rise to the right portal vein (RPV) at the PS level. The RPV has two bifurcations, the anterior (ARPV) and posterior (PRPV) branches (Fig. 1). The MPV is formed by the SpV and the SMV behind the pancreatic head and enters the liver in the porta hepatis, posterior to the hepatic artery and the common hepatic duct.

### Embryologic development of human UPVS

Three symmetric paired veins form the basis of the early venous system in 4th week of embryonic life, draining into sinus venosus: the UVs, vitelline veins (VVs) and cardinal veins (CVs) [2]. All three pairs open to the right and left horn of the sinus venosus. At between 4 and 6 weeks of embryonic life, a complex pattern of vessel growth, anastomosis and asymmetric degeneration occurs. The UVs course on either side of the septum transversum and the paired VVs pass through the septum transversum to the sinus venosus. The cranial segment of both veins between the liver and the heart is interrupted with an extensive vascular network-the hepatic sinusoids. The paired UVs form a “critical anastomosis” in the liver with the VVs and the hepatic sinusoids of the same side [3]. Two VVs create three anastomoses (cranial-ventral, dorsal and caudal-ventral anastomoses) with each other around the primitive foregut. They are named according to their anatomical position and relationship to the primitive foregut that will become the duodenum [4].

**Fig. 2:**
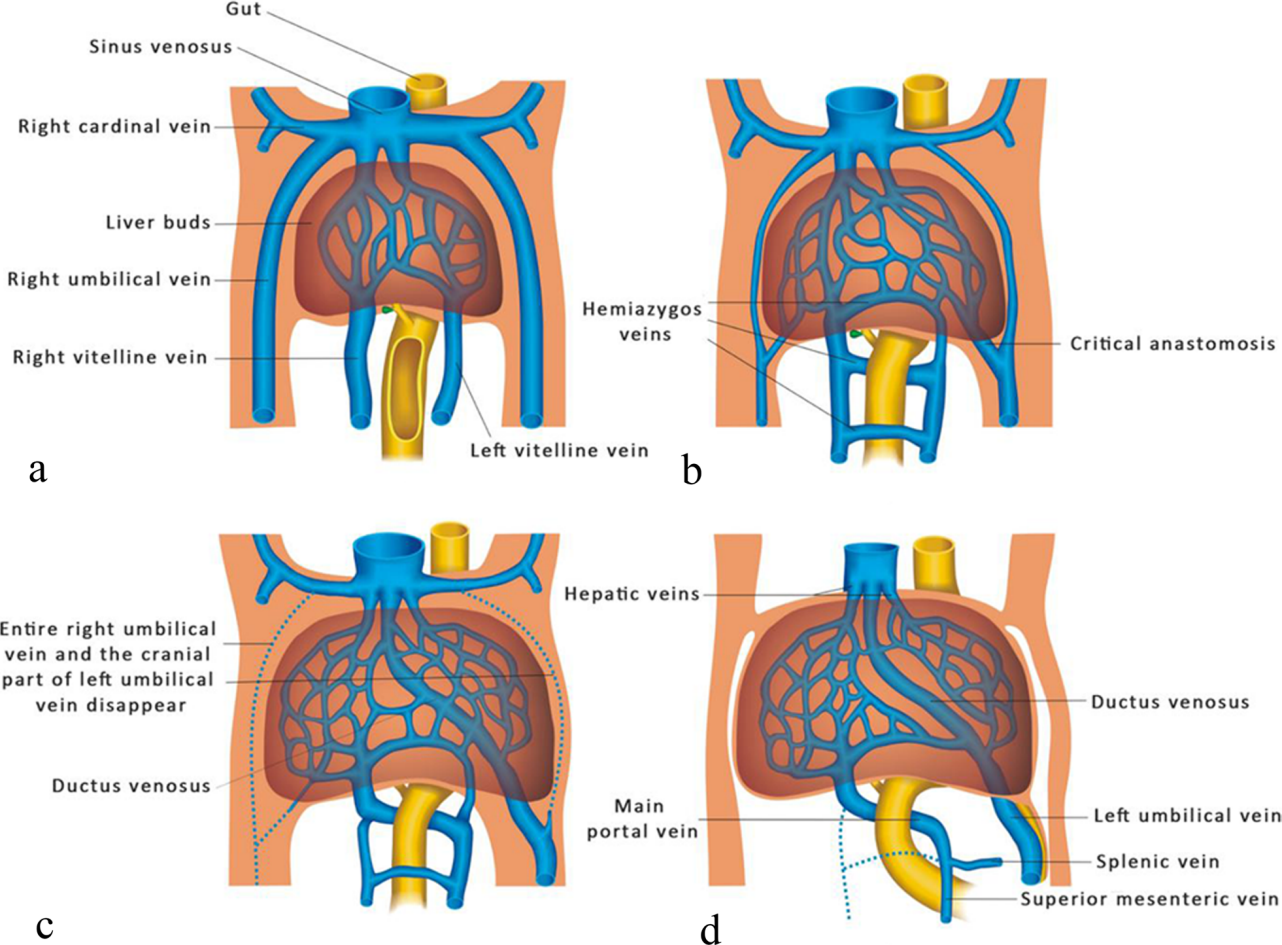
Embryological development of the human umbilical–portal venous system

By the 5th week of embryonic development, the caudal part of the right VV and a cranial part of the left VV progressively degenerate, and the remaining proximal right VV, which will give rise to the hepatocardiac segment of the IVC, is connected to the hepatic veins (HVs). Meanwhile, the dorsal anastomose become the PV. These changes in the VVs are accompanied by changes in the UVs. The entire right UV and the left cranial part of the left UV will disappear [2]. At the 8th week of development, the intrahepatic segment of the VV forms an anastomosis between the intrahepatic portion of the left UV and the DV, draining into the hepatocardiac segment of the IVC [5, 6] (Fig. 2).

### Previous classifications of congenital abnormalities of UPVS

Several classifications of congenital abnormalities of UPVS have been proposed from different perspectives. Morgan et al. [7] proposed the classification of portosystemic anomalies into two types based on whether or not the hepatic parenchyma is perfused with blood from the mesenteric venous system. Moore et al. [8] divided the abnormalities of the UV within the fetal abdominal which may be detected with prenatal sonography into three groups. In 2016, Achiron et al. [9] proposed the in utero classification which was based on the embryological–anatomical origin of the shunt, regarding the fetal venous system as a whole. This classification is the most comprehensive classification about fetal umbilical–portal–systemic venous shunt. However, it still cannot meet the clinical needs.

### New classification of congenital abnormalities of UPVS

In terms of identifying subtle anatomical or morphological anomalies, postnatal imaging examination (especially angiography) is better than prenatal ultrasonography in recognizing developmental abnormalities of UPVS. For ultrasonographists, mastering various congenital abnormalities of UPVS is of great importance for proper prenatal consultation and postnatal management. After analyzing numerous of prenatal ultrasonic imaging and literature, we proposed a relatively comprehensive classification of developmental abnormalities of UPVS. The new classification could identify three types: Type I, anatomy and morphological abnormalities of the PV; Type II, anatomy and morphological abnormalities of the UV; Type III, vascular connection abnormalities. Based on different prenatal sonographic manifestations, prognosis and perinatal management, all subtypes were classified into four prenatal diagnostic grading: necessary, amenable, difficult and unnecessary. The prenatal ultrasonographic manifestations, prenatal diagnostic grading, clinical findings and prognosis and perinatal management of each subtype were described in detail (Table 1).

**Table 1:** Ultrasonographic manifestations, diagnostic grading, prognosis and perinatal management of new classification of congenital abnormalities of UPVS

New classification	Subtypes		Prenatal ultrasonographic manifestations	Prenatal diagnostic grading	Clinical findings and prognosis	Perinatal management
Type I: Anatomy and morphological abnormalities of the portal vein	Preduodenal portal vein (PDPV)		Prenatal ultrasound may demonstrate duodenal obstruction as “double bubble” sign	Difficult	A majority of patients with PDPV are asymptomatic, but various clinical presentations and coexisting conditions can be present, the most common being duodenal obstruction	No special management is needed in the case with no clinical symptoms, and symptomatic work-up and treatment are required in the case with clinical symptoms, such as plain abdomen radiography examination and duodenoduodenostomy
	Portal vein and bile duct inverted variation		There is no report related to prenatal ultrasound diagnosis of the abnormality	Difficult	Most patients may have no obvious clinical symptoms, or mild abdominal distension or jaundice	No special management is needed in the case with no clinical symptoms
	Duplication of the portal vein (DPV)		There is no report related to prenatal ultrasound diagnosis of the abnormality	Difficult	The condition may be a cause of abdominal pain and can give rise to portal hypertension, with the development of esophagogastric varices, and may provide a source of fatal hemorrhage during childhood	No special management is needed in the case with no clinical symptoms, and symptomatic work-up and treatment are necessary in the case with clinical symptoms, such as postnatal ultrasonography and enhanced computed tomography
	Cavernous transformation of portal vein (CTPV)		There is no report related to prenatal ultrasound diagnosis of the abnormality	Difficult	Clinical symptom of CTPV can include portal hypertension, splenomegaly, ascites, gastrointestinal varices, obstructive jaundice, mesenteric venous congestion and ischemia, ascending cholangitis and biliary cirrhosis	Postnatal utrasonography or enhanced computed tomography can be performed for follow-up; the Rex bypass shunt is considered the gold standard strategy
	Portal vein stenosis or atresia		There is no report related to prenatal ultrasound diagnosis of the abnormality	Difficult	Portal vein obstruction, splenomegaly, variceal hemorrhage and portal hypertension could be the results of the anomaly; the prognosis is closely related to clinical symptoms	Postnatal utrasonography or enhanced computed tomography can be performed for follow-up, and symptomatic operative treatment are necessary
	Portal vein hypoplasia		There is no report related to prenatal ultrasound diagnosis of the abnormality	Difficult	Portal vein hypoplasia is a known comorbidity of biliary atresia, in addition to an enlarged hepatic artery; primary hypoplasia of the portal vein can cause secondary portal hypertension that presented with the severe but typical clinical manifestations of ascites and hepatic encephalopathy	Postnatal ultrasonography or enhanced computed tomography can be performed for follow-up, and symptomatic operative treatment and liver transplant evaluation is necessary
	Portal venous aneurysm (PVA)		Ultrasonography demonstrates dilatation of the PV system	Amenable	Most congenital cases are asymptomatic	Perinatal management primarily entails periodic surveillance of the aneurysm through medical imaging, Doppler ultrasonography is the most useful method
	Portal vein variants		Type 1—portal vein trifurcation, where the MPV divides into three branches: the LPV, the RAPV and the RPPV Type 2—the RPPV arises directly from the MPV as its first branch Type 3—the RAPV originates from the LPV Type 4—the portal vein gives only a single right portal branch in the liver hilum, and the left PV arises from the right anterior segmental branch	Unnecessary	Most patients are asymptomatic	No special management is required for isolated cases
	Transposition of the left and right portal vein		There is no report related to prenatal ultrasound diagnosis of the abnormality	Unnecessary	Most patients are asymptomatic	No special management is needed in the case with no clinical symptoms
Type II: Anatomy and morphological abnormalities of the umbilical vein	Intrahepatic persistent right umbilical vein (IPRUV)		The sonographic criteria included: (1) an aberrant course of the PV toward the stomach; (2) the fetal gallbladder being medial to the UV; (3) The UV fuses with the RPV instead of the LPV	Amenable	The prognosis of isolated intrahepatic PRUV has a very low risk for an adverse neonatal outcome; the prognosis of non-isolated cases depends on the severity of accompanied anomalies	An IPRUV should prompt an extended anatomic survey and a fetal cardiac evaluation; if the survey and cardiac anatomy are reassuring, no further follow-up is needed; if additional findings are identified, genetic counseling and invasive testing should be considered
	Duplication of the umbilical vein (DUV) (IPRUV)		Ultrasonography demonstrates two UVs in a transverse section of the fetal abdomen, which drain into the LPV and the RPV, respectively; the umbilical cord is also visible in four vessels	Amenable	Isolated DUV (intrahepatic PRUV) has a better outcome, and the prognosis of non-isolated cases depends on the severity of additional findings	A DUV (intrahepatic PRUV) should prompt an extended anatomic survey; no further follow-up is needed for isolated cases; if additional findings are identified, genetic counseling and invasive testing should be considered
	Umbilical vein varix (UVV) (normal direction)		Sonographically, UVV appears as whole course ectatic anechoic UV or limited mass, and color Doppler sonography detects the bidirectional turbulent flow, at the level of the dilated segment of the umbilical vein	Amenable	The prognosis of isolated case has a very low risk for an adverse neonatal outcome	A systematic structural examination should be performed; no further follow-up is needed for isolated cases; genetic testing is recommended in the fetus with non-isolated UVV
	Umbilical vein constriction		Prenatal ultrasound shows the diameter of the UV is narrower than the normal range, and pulsed Doppler detects high-speed blood flow, which can be as high as 150 ~ 200 cm/s	Necessary	The pregnancy complications include IUFD, IUGR and oligohydramnios	Close fetal ultrasonographic surveillance is necessary
Type III: Vascular connection abnormalities	Portal-systemic shunts (PSS)	IHPSS	The prenatal ultrasound is shown the connection with the IHPVS and the HV, and the DV should be focused	Necessary	Fetuses with IHPSS have the highest live birth rate compared to other types of shunt fetuses, which can be naturally closed after delivery	Hemodynamic surveillance should be performed in fetal period; for the cases cannot be closed naturally, surgery was performed to repair the shunt
		EHPSS	The antenatal ultrasound visualizes the SpV and the SMV shunting into the IVC	Necessary	The prognosis of EHPSS depends on the size of shunt volume, the present of associated malformations, and the development of hemodynamic imbalance; the prognosis of most cases of the complete absence of the IHPVS are poor	Hemodynamics should be closely monitored before delivery
	Umbilical–systemic shunts (USS)		The prenatal ultrasound detects that the UV directly connects to the systemic circulation, such as the right atrium, the IVC, the renal vein, or the iliac vein	Necessary	USS is often associated with deficiency of the DV and dysplasia of portal venous system; the risk of chromosome abnormality and other structure malformations is high; fetuses with USS have the poorest prognosis, and the lowest rates of live birth and postnatal survival are observed	Given the high prevalence of congestive heart failure and edema, hemodynamic surveillance should be performed in fetal period
	Ductus venosus-systemic shunts (DVSS)		Prenatal ultrasound shows abnormal shunt between the DV and the abdominal IVC, the hepatic vein or the coronary sinus	Necessary	The fetuses with the DVSS are associated with a high incidence of chromosomal malformation and a low risk of other structural malformations; fetuses with isolated DVSS have a good prognosis and normal liver function	Genetic examination should be recommended for fetuses with DVSS detection to exclude chromosomal abnormalities
	Congenital hepatoportal arteriovenous fistula (CHPAVF)		Prenatal ultrasound shows single or multiple direct communications between the hepatic artery and the portal vein branches; additional findings include hepatic artery enlargement, portal vein dilatation at the site of fistula and abdominal aorta tapering beyond the celiac artery	Necessary	The CHPAVF can lead to high out-put heart failure with a mortality rate of 50% ~ 90%	The prenatal diagnosis of CHPAVF enables better planning of postpartum management
	Isolated absence or atresia of ductus venosus		Prenatal ultrasound demonstrates that the DV is absent or presents as a thin band connecting the LPV and the IVC, and that CDFI examination could not detect the blood flow signal	Necessary	Isolated absence or atresia of DV had good prognosis in 67.2% cases, and died in perinatal period as a result of fetal edema in 15.6% cases	Close surveillance of fetal hemodynamic changes is recommended
	Abnormal entry of the umbilical vein into the portal vein		Prenatal ultrasound shows the UV connecting with the MPV after entering the abdominal cavity, and the confluence of the UV and the MPV presents as an aneurismal dilatation. There is a variety of variations of influent blood vessel at the joint, most are the SMV and the SV	Necessary	Prognosis is depended on thrombus formation, a persistent portal thrombosis can cause portal cavernoma with portal hypertension that led to esophageal varices	Sonographic surveillance and amniocentesis was counseled; close postpartum ultrasonographic follow-up is necessary; the case without thrombosis can be conservatively observed and followed up; if a thrombosis is suspected, early surgery is proposed in order to avoid persistent portal thrombosis and its specific complications

### Anatomy and morphological abnormalities of the portal vein

#### Preduodenal portal vein

**Fig. 3:**
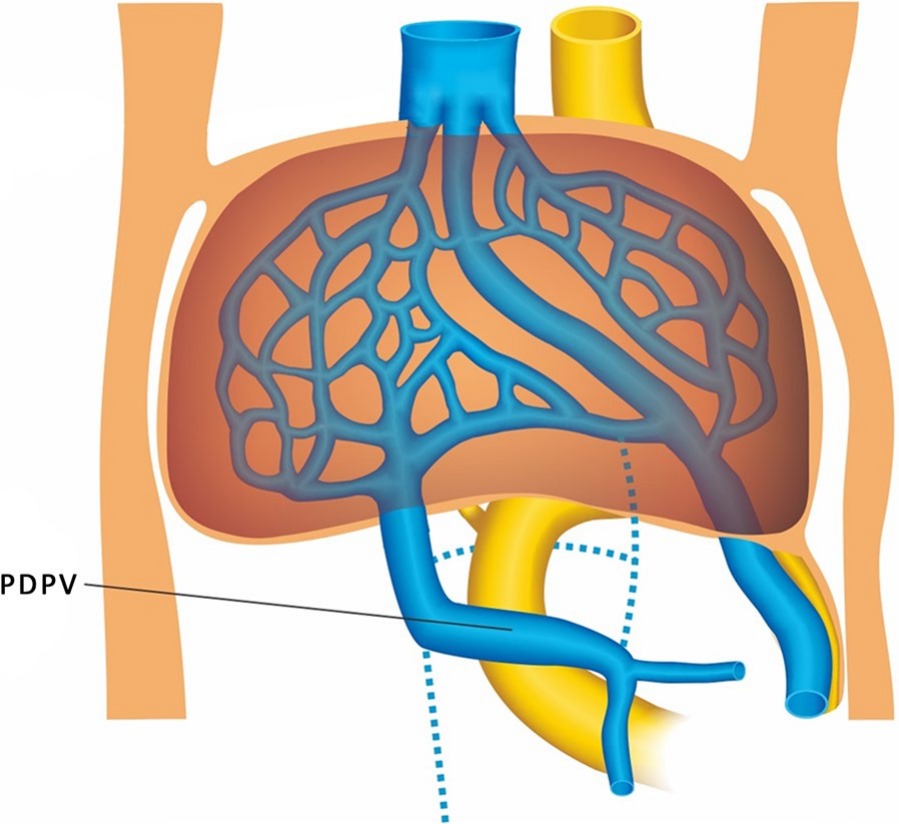
The embryological scheme of PDPV. At the embryonic development, dorsal anastomose of the VVs degenerates and caudal-ventral anastomose persists forming PDPV

Preduodenal portal vein (PDPV) is a rare congenital anomaly in which the vein passes anteriorly rather than posteriorly to the duodenum. This congenital anomaly was first described by Knight in 1921 [10]. At the embryonic development, dorsal anastomose of the VVs degenerates and caudal-ventral anastomose persists forming PDPV (Fig. 3). A majority of patients with PDPV are asymptomatic, but various clinical presentations and coexisting conditions can be present, the most common being duodenal obstruction [11]. It may cause duodenal obstruction by directly compressing the lumen of the duodenum or the associated anomalies may cause duodenal obstruction [12]. Duodenal obstruction caused by PDPV usually requires surgical treatment. PDPV usually associated with other anomalies such as malrotation of gut, annular pancreas, biliary malformation, splenic anomalies and situs inversus. A majority of PDPV-related reports are pediatric patients. Choi and Park [13] reported a case of duodenal obstruction diagnosed by prenatal ultrasound in 1995. The postoperative diagnosis was PDPV and intestinal malrotation after birth. This case provides a new idea for prenatal ultrasound diagnosis of “double bubble” sign. Grate attention should be paid to the location of the fetal PV to confirm the possibility of PDPV.

#### Portal vein and bile duct inverted variation

The anatomical structure of the normal first hepatic portal in the order from front to back is the extrahepatic bile duct, the proper hepatic arteria, the PV. When the PV and the bile duct are inverted, the extrahepatic bile duct is located deep behind the PV. It may be related to the abnormal position of VVs and hepatic diverticulum during embryonic development. Most of the variation was found in surgical exploration. There is no report related to ultrasound diagnosis of this variation prenatally and postnatally. Prenatal ultrasound is difficult to detect the extrahepatic bile duct and cannot make a definite prenatal diagnosis. It has been reported that dilated common bile duct is presented in most adult cases. The ultrasonographic manifestation presents the double duct sign in the porta hepatis. The portal vein is located in front of the common bile duct and could be distinguished by color Doppler. Most patients may have no obvious clinical symptoms, or mild abdominal distension or jaundice.

#### Duplication of the portal vein

**Fig. 4:**
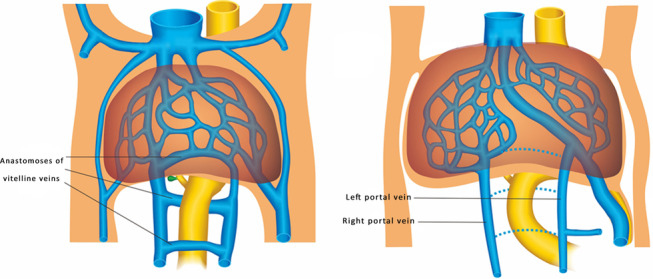
The embryological scheme of DPV. At the embryonic development, the dorsal and caudal-ventral anastomose of the vitelline veins degenerate, and the caudal part of the two vitelline veins persist

Duplication of the portal vein (DPV) is an uncommon malformation, which can be divided into three types: extrahepatic DPV, intrahepatic DPV and double sagittal part. It has been reported that the pathogenesis of DPV may be related to the persistence of the caudal part of the left VV and the abnormal degeneration of the dorsal anastomose of the VVs (Fig. 4). The location relationship between the two PVs and the duodenum is variable [14–16]. The congenital abnormality may give rise to portal hypertension, with the development of esophagogastric varices, and may provide a source of fatal hemorrhage during childhood [15]. In the case of the PV(s) in front of the duodenum may predispose to digestive tract obstruction and the abdominal pain, symptomatic treatment is recommended.

#### Cavernous transformation of portal vein

Cavernous transformation of portal vein (CTPV) refers to collateral vessel formation around the PV and/or its tributaries after completely or partially blocked, or appearance as a kind of special spongy after the PV recanalized. It is a kind of compensatory lesion to ensure liver blood flow and function, and its main complication is chronic portal hypertension.

CTPV can be divided into congenital and secondary categories. The congenital CTPV refers to the congenital dysplasia of the PV or the extension of the UV involutes after birth, which makes the PV stenosis or even atresia. Clinical symptom of CTPV can include portal hypertension, splenomegaly, ascites, gastrointestinal varices, obstructive jaundice, mesenteric venous congestion and ischemia, ascending cholangitis and biliary cirrhosis [17]. Although recurrent upper gastrointestinal hemorrhage and peritoneal effusion, the liver function is normal. Ultrasonography demonstrates that the portal bifurcation may be replaced by an echogenic structure with multiple small tortuous vessels. The portal trunk may appear as a network of vessels or as slender tortuous vessels within an echogenic structure [18]. However, there is no better treatment for the formed vascular malformations. Drugs, interventions and surgical procedures can be used to prevent or treat bleeding. Surgical procedures include devascularization, shunt, combined surgery and liver transplantation, of which shunt is the most commonly used method [19]. The long-term prognosis depends on the severity of the associated abnormalities.

#### Portal vein stenosis or atresia

Stenosis or atresia of the PV may involve all or a portion of the PV. The UV drains into the LPV in embryonic period and spontaneously involute at birth. If this obliterative process is excessive, PV atresia and/or stenosis can develop [15]. Portal vein obstruction, splenomegaly, variceal hemorrhage and portal hypertension could be the results of such anomaly [15]. Atresia of a major branch of the PV can have the associated absence of the corresponding hepatic lobe. Congenital complete atresia of the PV may involve extrahepatic portosystemic shunt of splenomesenteric vein system [20].

#### Portal vein hypoplasia

The PV, which is as small as or smaller than the adjacent hepatic artery, can be generally considered as hypoplasia of the PV [21]. Congenital portal vein dysplasia is thought to be secondary to development failure of the PV and/or its branch or embryonic thrombogenesis affects the development of the corresponding hepatic lobe or segment. The corresponding hepatic lobe may be atrophic as a result of the dysplastic PV. Hypoplasia of the PV can be depicted in children with biliary atresia, in addition to enlargement of the hepatic artery. PV hypoplasia in the setting of biliary atresia has an incidence of 26% [21]. This increases the risk of complications associated with liver transplantation, strongly linked to the risk of thrombosis [20, 21].

#### Portal venous aneurysm

**Fig. 5:**
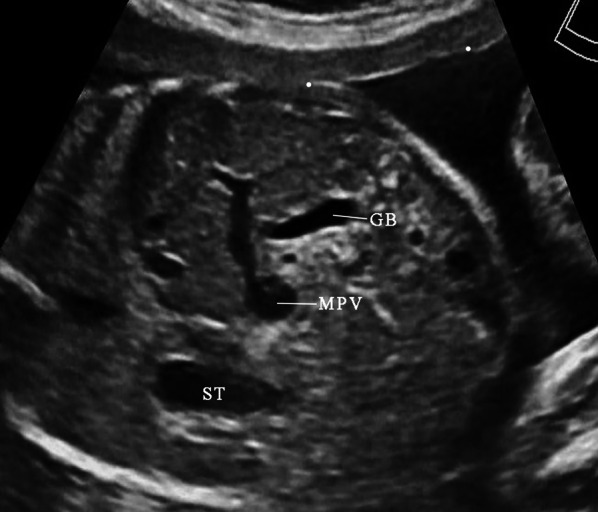
Grayscale ultrasound images showing fetal portal vein aneurysm. In utero abdominal plane demonstrating focal dilatation of the MPV

Portal vein aneurysm (PVA) is an unusual vascular dilatation of the portal vein with an incidence of 0.06%, accounting for 3% of venous aneurysms [22, 23]. The most common sites at which portal venous system aneurysms develop are the main portal vein and the confluence of the splenic and the superior mesenteric veins. Extrahepatic aneurysms are more common than intrahepatic aneurysms [24]. It is anticipated that the failure of regression of the right primitive vitelline vein leads to congenital aneurysms of the portal vein and develops a diverticulum from the vitelline vein remnants [25]. When signs and symptoms do not suggest acquired causes, a congenital etiology is assumed. Congenital PVA can be diagnosed using ultrasound in utero [26] (Fig. 5).

Most patients are usually asymptomatic as PVA is an incidental finding, especially for small PVA. Large PVA can cause epigastric or right hypochondriac pain, gastrointestinal bleeding, obstructive jaundice or gastric outlet obstruction, portal vein thrombosis and aneurysmal rupture [24]. Ultrasonography has been used for the prenatal and postnatal evaluation and diagnosis of PVA, and shows focal dilatation of the portal vein system. Pulsed Doppler image of the aneurysm can demonstrate venous flow. Thrombosis can be diagnosed with the use of Doppler by the absence of flow in the vessel.

Most asymptomatic patients primarily entail periodic surveillance [23]. Surgical treatments differ according to the presence of portal hypertension. In patients without portal hypertension, aneurysmorrhaphy or aneurysmectomy are recommended, while surgical shunt procedures or liver transplantation are demanding options for patients with portal hypertension [23].

#### Portal vein variants

**Fig. 6:**
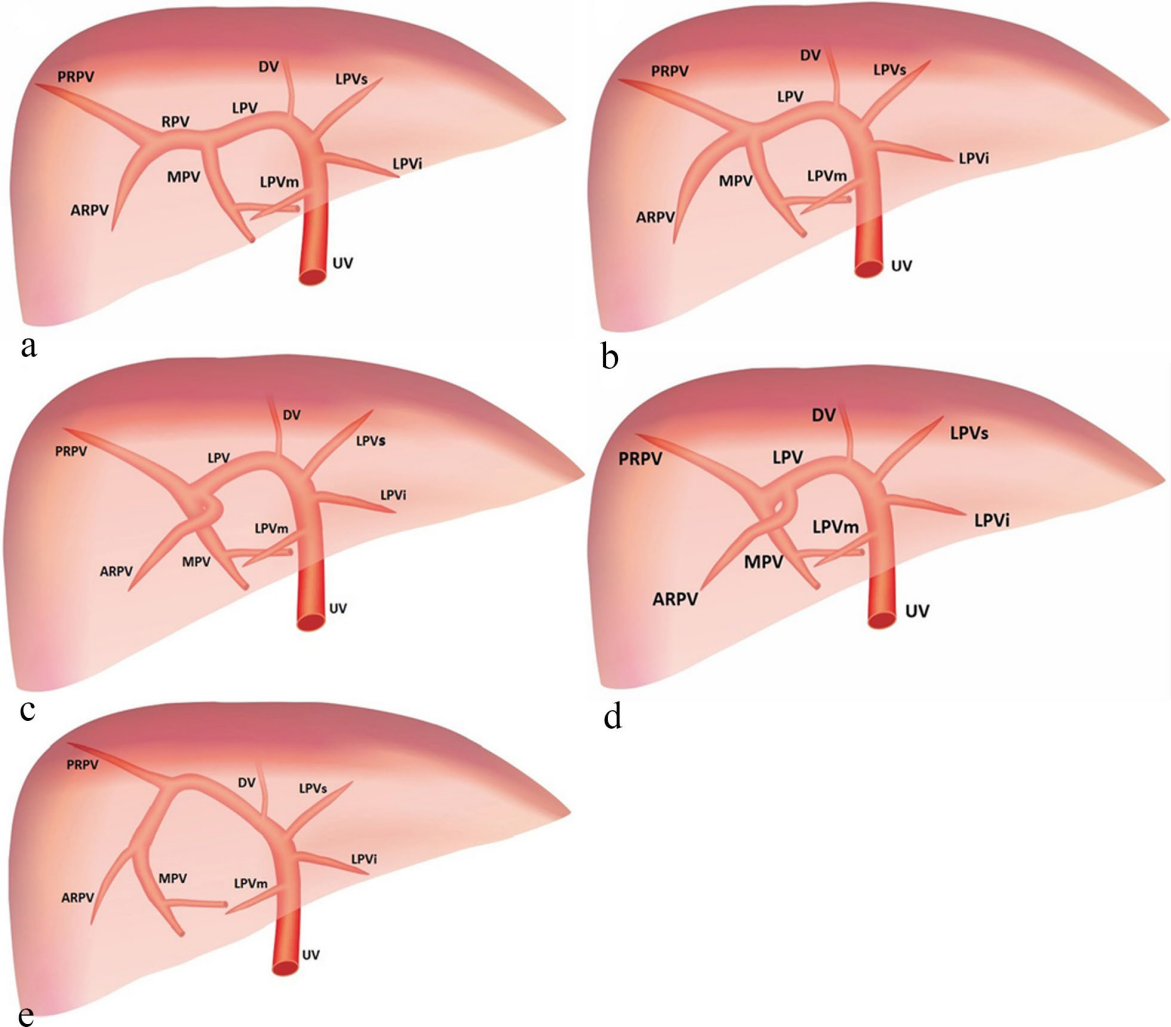
Schemes illustrate the normal anatomy and anatomic variants in the connections of the intrahepatic portal veins. The normal course of the intrahepatic portal veins (**a**). Different types of portal vein variants: Type 1 (**b**), Type 2 (**c**), Type 3 (**d**) and Type 4 (**e**)

**Fig. 7:**
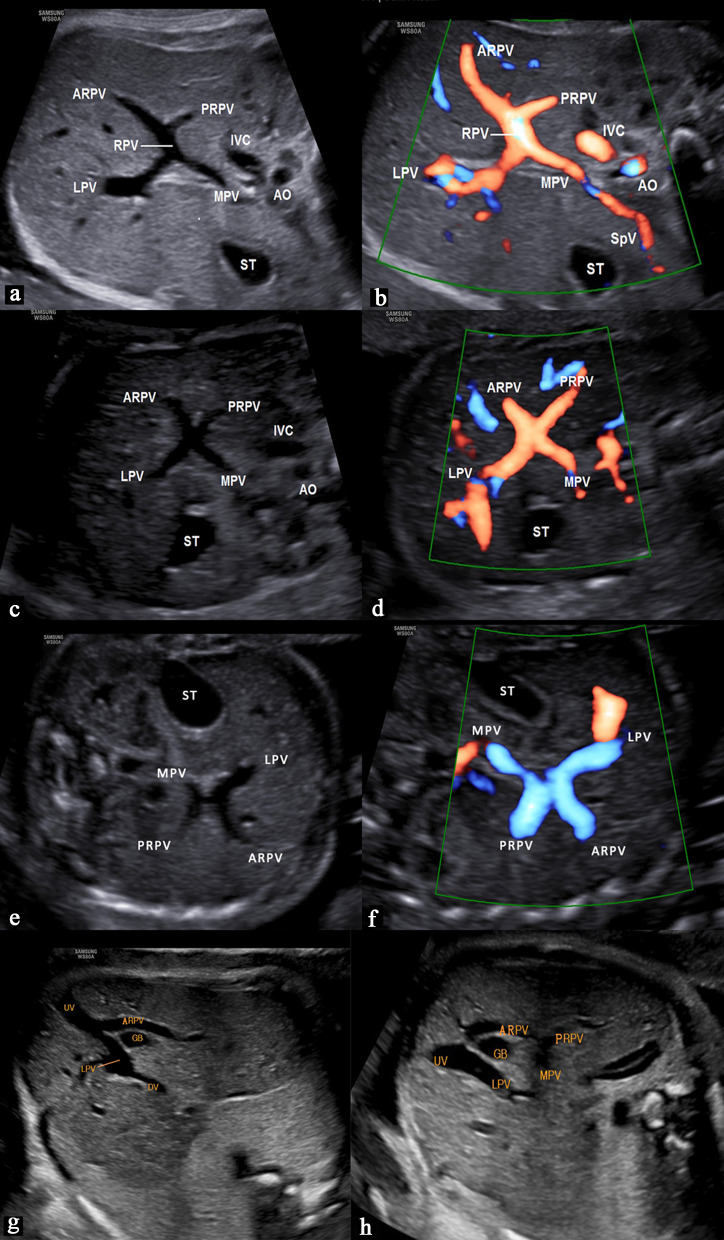
Sonographic images of fetal normal connection and anatomic variants of the intrahepatic portal veins. The normal course of the intrahepatic portal veins (**a**, **b**). Different types of portal vein variants: Type 1, portal vein trifurcation (**c**, **d**); Type 2, the PRPV originates as the first branch of MPV (**e**, **f**); Type 3, the ARPV originates from the LPV (**g**, **h**)

The normal MPV divides the liver hilum into two branches: the LPV branch and the RPV branch. The RPV branch divides secondarily into two branches: the ARPV and the PRPV. The LPV runs horizontally to left, then turns medially (Figs. 6a, 7a, b). This standard branching pattern was observed in approximately 70–80% of the population [27].

Four main types of portal vein variants are described [27–29]:

- Type 1: portal vein trifurcation, where the right anterior, right posterior, and left portal branches arise from the same point with a reported occurrence of 9–11% (Figs. 6b, 7c, d).
- Type 2: right posterior branch arising as the first branch of main portal vein within the hepatic hilum, the occurrence is reported 1–7% (Figs. 6c, 7e, f).
- Type 3: the ARPV originates from the LPV was observed in 1–6% of population (Fig. 6d, 7g, h).
- Type 4: the portal vein gives only a single right portal branch in the liver hilum, and the left PV arises from the right anterior segmental branch. This type is less common (Fig. 6e).

#### Transposition of the left and right portal vein

Transposition of the left and right portal vein is a rare development abnormality of the PV, mostly discovered during surgery and associated with situs inversus. Patients without other abnormalities are usually asymptomatic, and liver function mostly are normal. Ultrasonography shows that the LPV and the branches are located in the middle of the hepatic right lobe. The original left superior segmental branches course in the hepatic right posterior lobe, and the left inferior segmental branches course in the right anterior lobe, while the left medial lobal branches still lie in the left medial lobe. The RPV presents as a Y-shaped vein, of which the bifurcation distributes in the superior segment and inferior segment of the left lateral lobe.

### Anatomy and morphological abnormalities of the umbilical vein

#### Intrahepatic persistent right umbilical vein

**Fig. 8:**
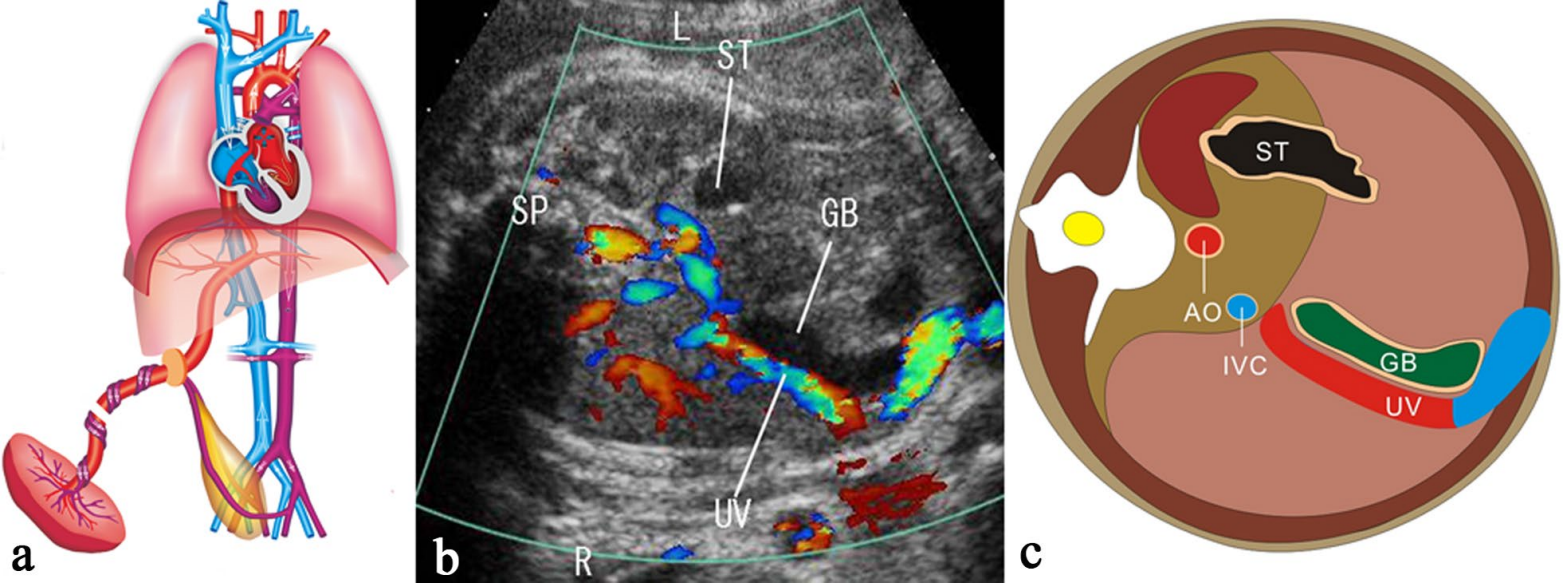
The scheme (**a**, **c**) and color Doppler flow imaging (**b**) of intrahepatic PRUV. The cross sections of fetal abdomen (**b**, **c**) show the RPV turning toward the ST, connecting with a PRUV

Persistent right umbilical vein (PRUV) is an altered embryonic development, in which the LUV regresses and the RUV remains open. In the intrahepatic variant, the umbilical vein fuses with the right portal vein, and placental blood continues to the DV, eventually draining into the IVC [30] (Fig. 8). It is reported that the overall prevalence of intrahepatic PRUV is 0.13% [31]. Typically, PRUV is an isolated anomaly; however, it may be accompanied by other disorders in the cardiovascular, neurological or genitourinary systems [32]. In patients with intrahepatic PRUV, a thorough extended anatomic survey should be performed. Extra malformations provide strong evidence to recommend a genetic testing. The prognosis of isolated intrahepatic PRUV has a very low risk for an adverse neonatal outcome, no further testing is needed.

#### Duplication of the umbilical vein (intrahepatic PRUV)

**Fig. 9:**
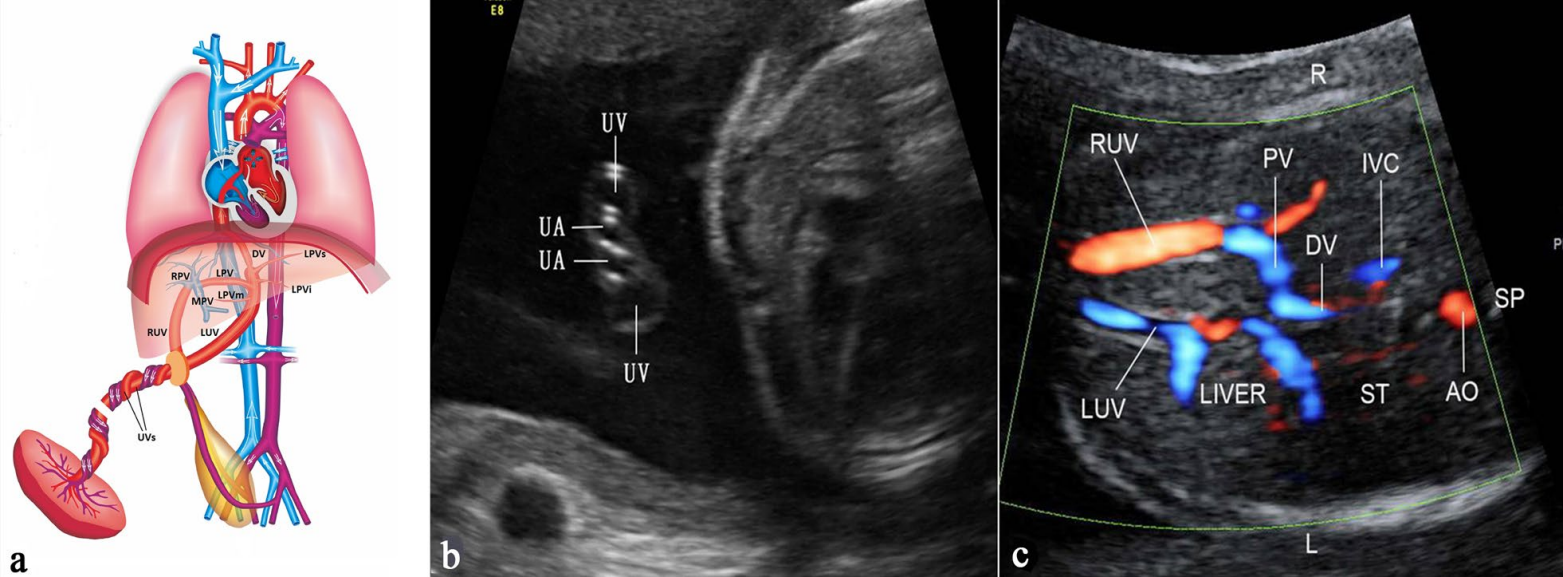
The scheme (**a**) and ultrasound images (**b**, **c**) of DUV (intrahepatic PRUV)

Duplication of the umbilical vein (DUV) is an extremely rare finding referring as an increase in the number of vessels to four (two arteries and two veins). DUV is a result of the LUV and RUV both open instead of the RUV degenerates. In most cases, ultrasonography shows that the RUV enters the liver and connects with the RPV, and the LUV usually merges with the LPV and drains into the IVC through the DV as a normal course (Fig. 9). The patient with isolated DUV (intrahepatic PRUV) has a better outcome, and none of the special treatment is needed after birth. Previous reports have described that DUV may be associated with cardiovascular, neurological or facial systems malformations [33, 34], of which the prognosis depends on the severity of associated malformations, and genetic testing is recommended.

#### Umbilical vein varix (normal direction)

**Fig. 10:**
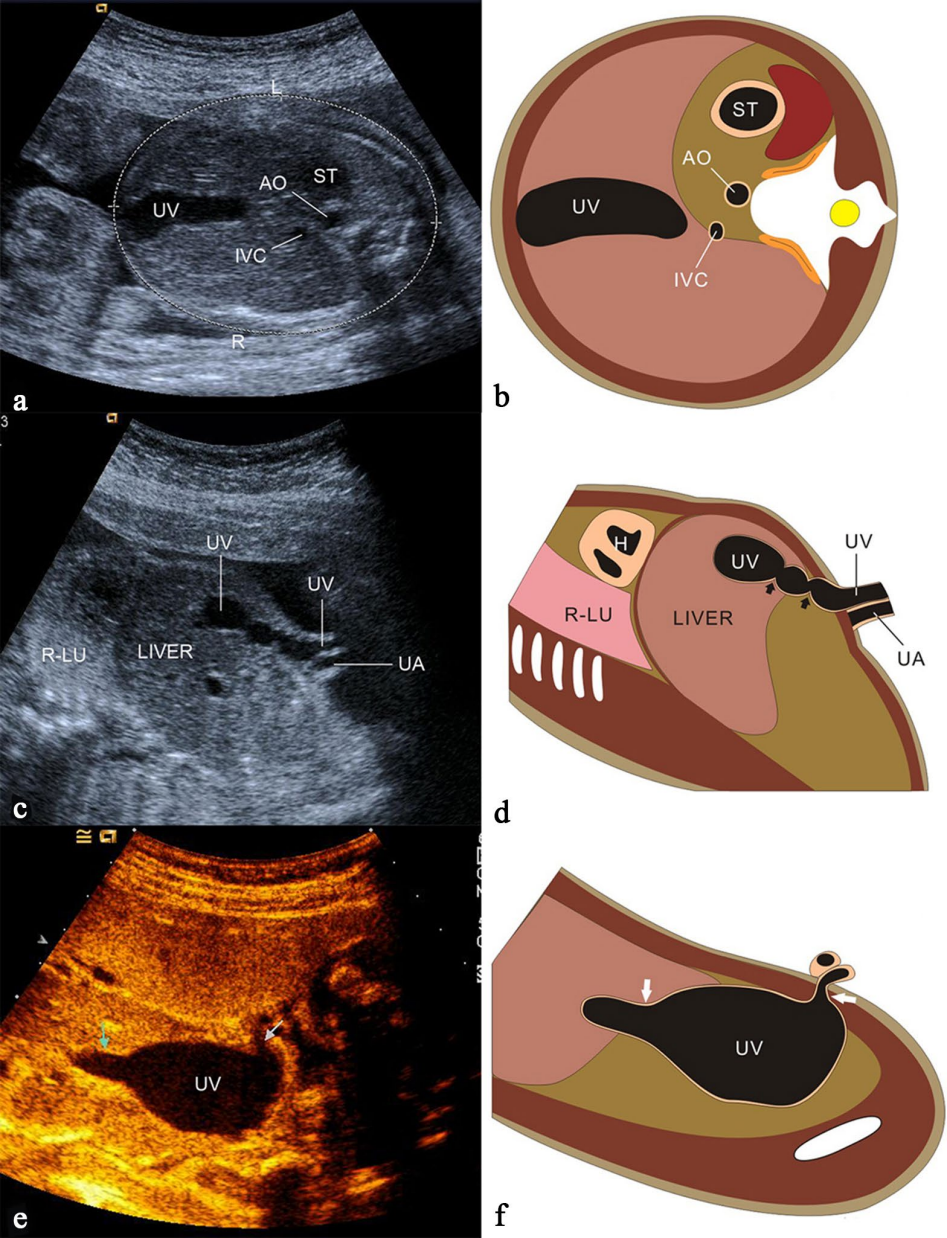
Grayscale ultrasound image and scheme of the fetus showing different types of UVV. Images **a**, **b** illustrate the whole course dilation of UV. Images **c** and **d** show the multiple limited UVV. Images e and f show UVV like the aneurysm

Umbilical vein varix (UVV) accounts for 4% of the malformations of the umbilical cord in the fetus with an incidence of 0.4–1.1/1000 [35]. UVV was defined as a portion of umbilical vein that is at least 50% wider than the non-dilated portion, a dilatation of ≥ 9 mm or dilatation of > 2SD above the mean value for gestational age [36]. The weak supporting structure of the UV contributes to the formation of UVV [37]. Sonographically, UVV appears as whole course ectatic anechoic UV or limited mass (Fig. 10), and color Doppler sonography detects the bidirectional turbulent flow, at the level of the dilated segment of the umbilical vein [35]. The main complications of UVV are intrauterine fetal demise (IUFD), thrombosis and intrauterine growth restriction (IUGR) with the total incidence of 10% [35]. The fetuses with isolated UVV have a very low likelihood of having associated chromosomal anomalies [36]. While compared with fetuses with isolated UVV, the incidence of chromosomal anomalies and the risk of IUFD for non-isolated UVV is 15-fold and eightfold, respectively [36]. A systematic structural examination should be performed, especially for the fetus with UVV diagnosed in the early pregnancy, and genetic testing is recommended in the fetus with non-isolated UVV.

#### Umbilical vein constriction

**Fig. 11:**
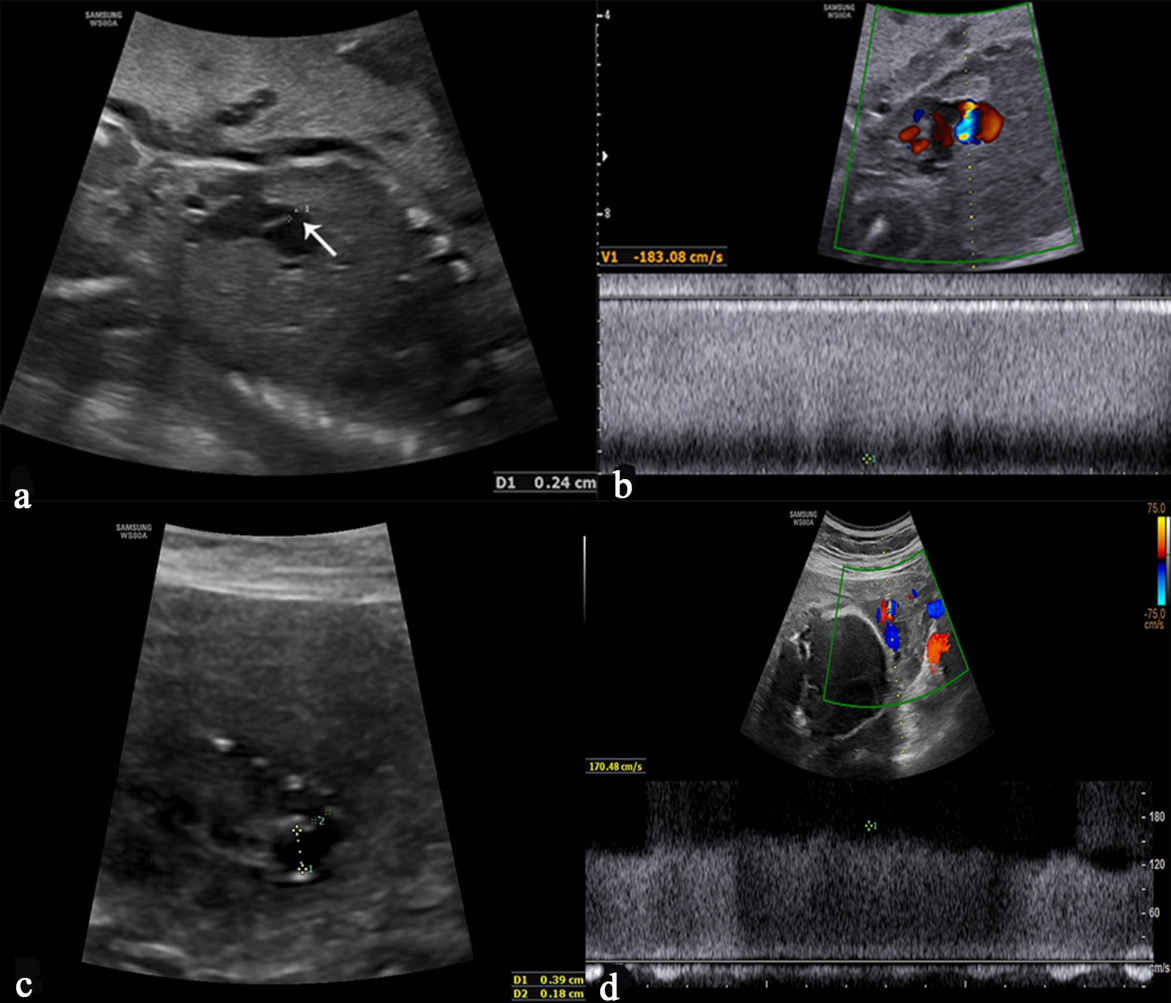
Grayscale ultrasound image and pulsed Doppler image demonstrate the constriction of the umbilical vein. Images **a** and **b** illustrate the membranous constriction of the umbilical vein (arrow) in the intra-abdominal section. Images **c** and **d** show constrictive umbilical vein at a free loop

In low-risk fetuses, the mean inner diameter of the vein in the cord is 3.6–8.2 mm (mean 13–19 cm/s) during gestational weeks 20–40 while the corresponding diameter at the umbilical ring is less at 2.8–5.9 mm (mean 34–41 cm/s) [38]. The diagnostic criteria of UV constriction are the inner diameter of the UV narrower than the mean diameter of corresponding gestational weeks and the venous blood velocity could be up to 150–200 cm/s (Fig. 11). UV constriction is associated with pregnancy complications, including IUFD, IUGR and oligohydramnios. The UV constriction causes a decrease in the blood flow to the fetus. If the blood flow is decreased enough to be unable to meet the demands of the developing fetus, the fetus develops IUGR, becomes hypoxic and then acidotic, and IUFD [39]. Close fetal surveillance enabled early detection of abnormal fetal heart rate tracing, which may have prevented IUFD [40]. Careful assessment of UV constriction may be necessary to prevent poor perinatal outcomes.

### Vascular connection abnormalities

#### Portal-systemic shunts

Achiron and Kivilevitch [9] proposed the anatomical–clinical classification of fetal umbilical–portal–systemic venous shunt in which portal-systemic shunt (PSS) is further divided into two subtypes: intrahepatic portal-systemic shunt (IHPSS) and extrahepatic portal-systemic shunt (EHPSS).

**Fig. 12:**
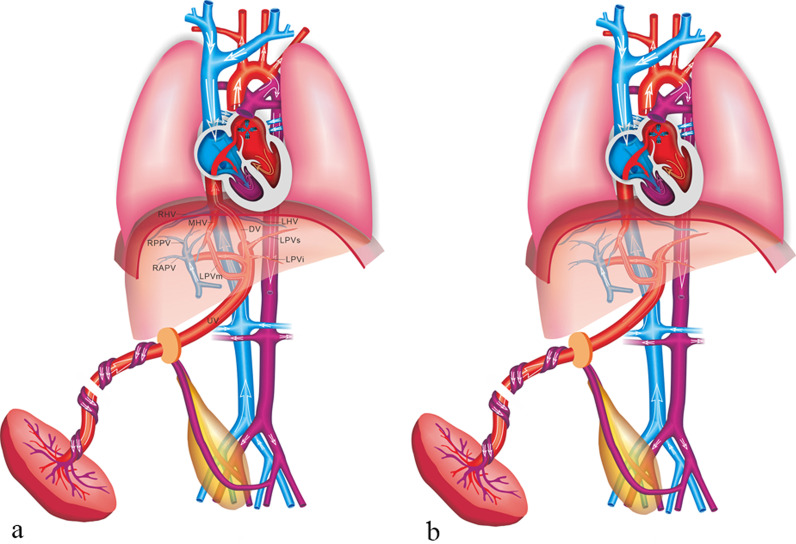
Schematic drawing of the IHPSS with the DV presence (**a**) and absence (**b**)

**Fig. 13:**
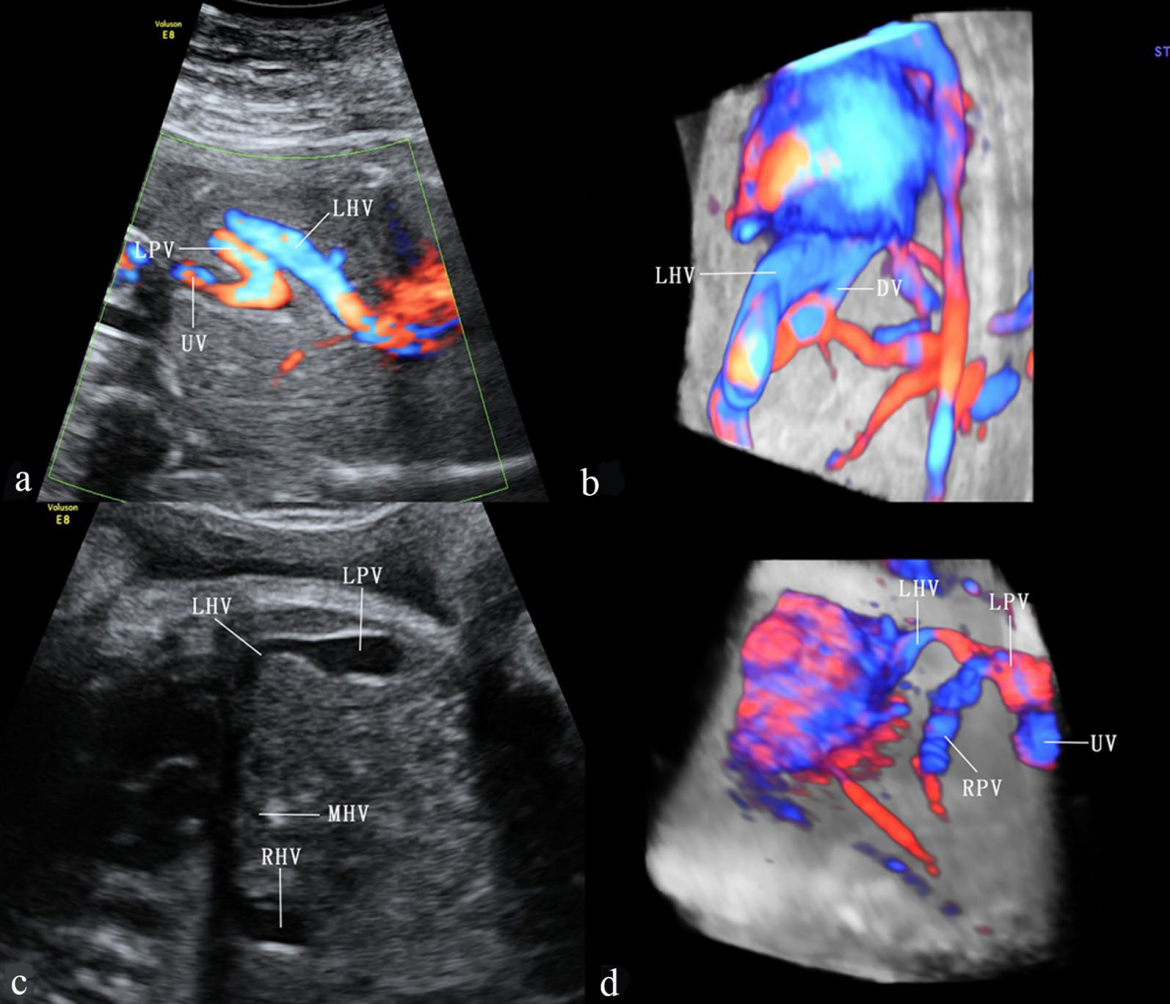
High-definition power flow Doppler image, 3D high-definition power flow Doppler image and grayscale ultrasound image show unilateral shunt between the LPV and the LHV with the DV presence (**a**, **b**) and absence (**c**, **d**)

IHPSS is an anastomosis between the HVs and intrahepatic portal venous system (IHPVS). The DV and IHPVS could be intact or absent [9] (Figs. 12, 13). The case with IHPSS has a high risk of IUGR, which should focus observation by prenatal ultrasonography. It has been shown that IHPSS fetuses have the highest live birth rate compared to other types of shunt fetuses, which can be naturally closed after delivery [9, 41]. Early hemodynamic surveillance should be performed in fetal period. The liver enzyme and serum ammonia level of the neonates should be monitored, and venography may be considered to verify the IHPSS. For the cases cannot be closed naturally, surgery was performed to repair the shunt.

**Fig. 14:**
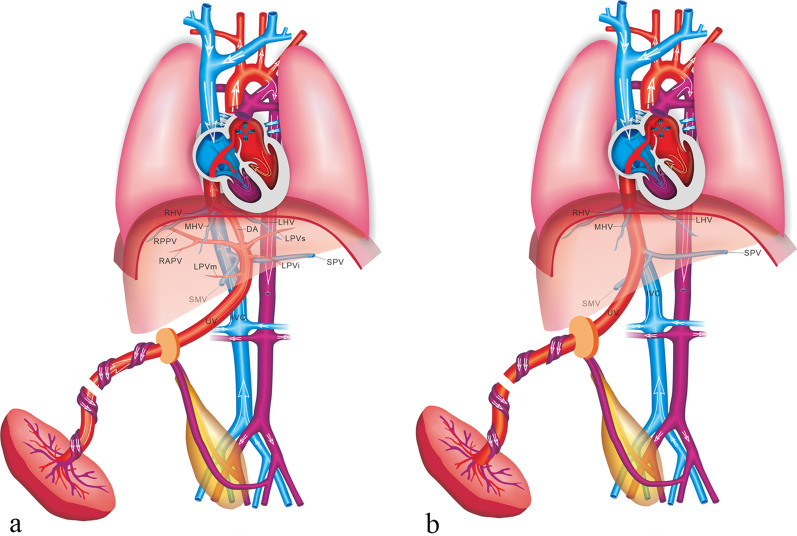
Schematic drawing of the EHPSS. The MPV drains into the IVC with the IHPVS presence (**a**) and absence (**b**)

The EHPSS is characterized by the diversion of the portal blood into the vena cava, with complete or partial absence of IHPVS (Fig. 14). The case with EHPSS can involve multiple structural malformation, especially those with the complete absence of IHPVS [42]. The prognosis of EHPSS depends on the size of shunt volume, the present of associated malformations, and the development of hemodynamic imbalance with signs of heart failure, cardiomegaly and hydrops [43]. Hemodynamic changes should be closely monitored in utero, and the fetus should be delivered as soon as possible with the sign of heart failure. Intrahepatic portal venous perfusion insufficiency can lead to abnormal liver development, abnormal liver function and abnormal hyperplasia. The risk of liver malignant tumor in such patients is high. Other complications included neonatal cholestasis, hepatopulmonary syndrome, encephalopathy, and pulmonary hypertension might be consequences of the EHPSS [43]. Surgical management can help patients relieving symptoms and preventing complications, including surgical closure, interventional embolization and liver transplantation [44].

#### Umbilical–systemic shunts

**Fig. 15:**
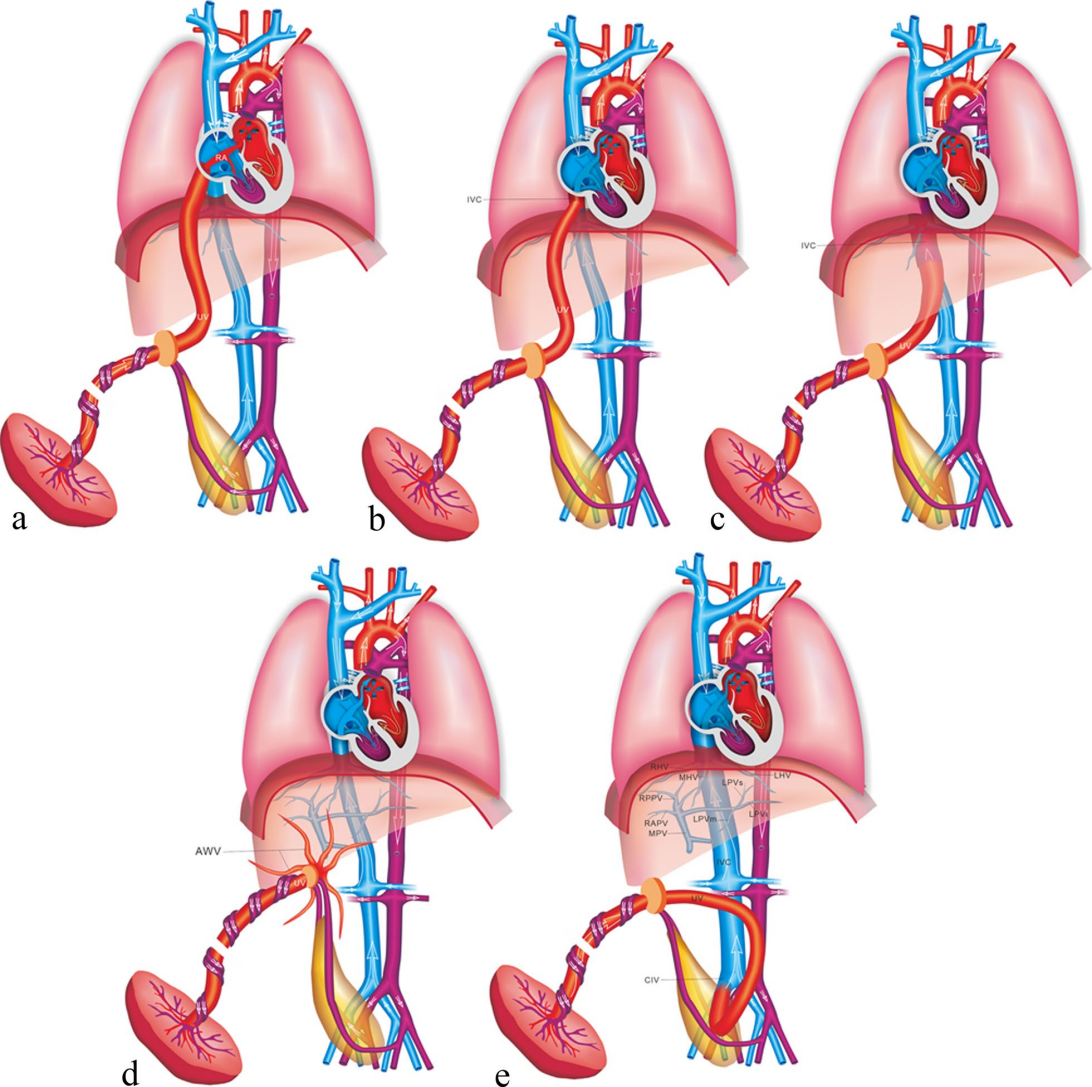
Schematic drawing of the USS. The UV directly connects to the right atrium (**a**), the intra-thoracic IVC (**b**), the abdominal IVC (**c**), the AWV (**d**), and the CIV (**e**)

In the case of umbilical–systemic shunts (USS), the UV failed to form the normal intrahepatic connection with the LPV–DV, due to agenesis of both the LPV and the DV [9], and directly connected to the systemic circulation, such as the right atrium, the IVC, the renal vein or the iliac vein (Fig. 15). USS is often associated with deficiency of the DV and dysplasia of portal venous system. The risk of chromosome abnormality and other structure malformations in such case is high. Previous studies showed that the USS was characterized by the highest incidence of the complete absence of normal IHPVS and the highest incidence of associated major anomalies [9, 41]. The fetuses with USS have the poorest prognosis, and the lowest rates of live birth and postnatal survival are observed [41].

#### Ductus venosus-systemic shunts

**Fig. 16:**
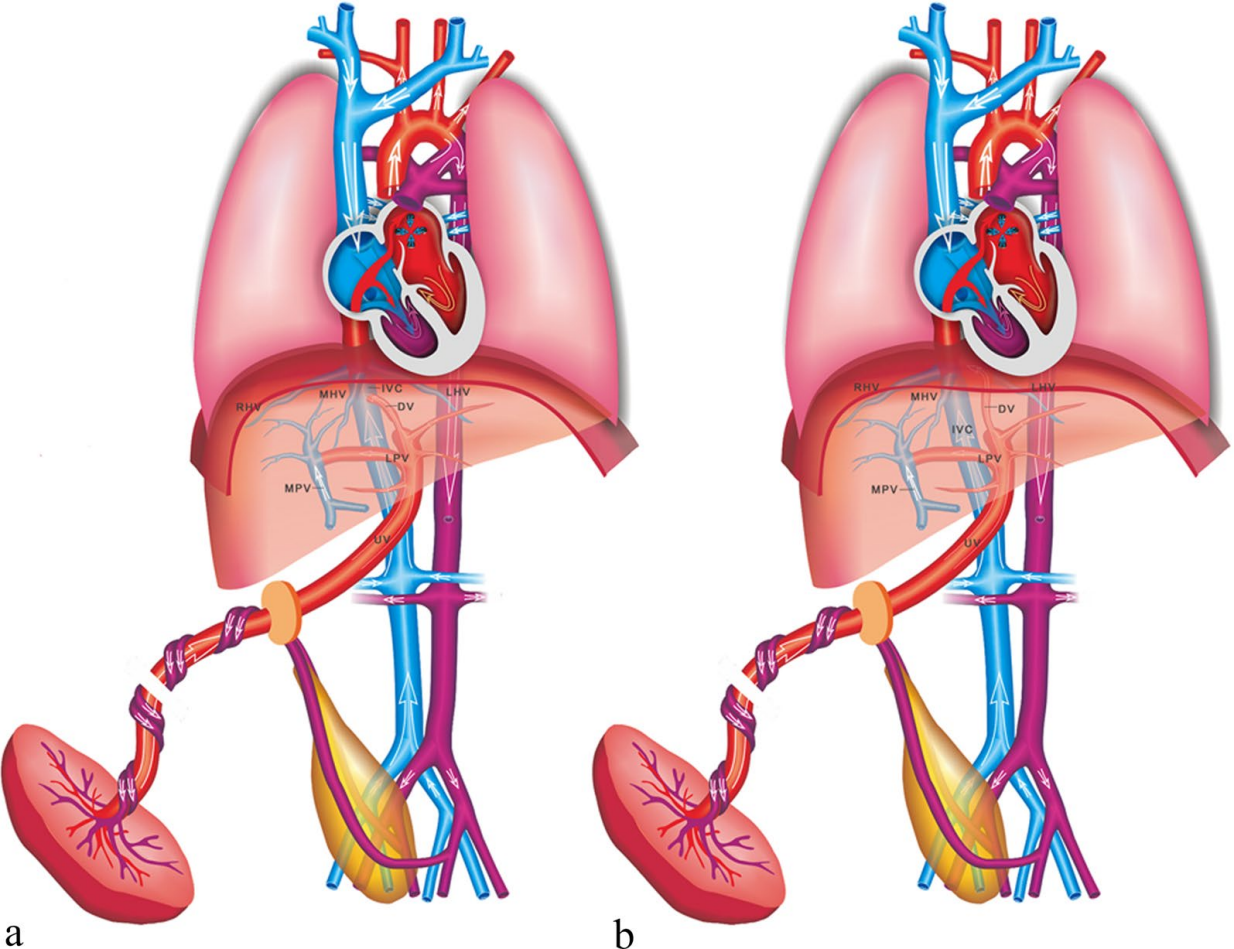
Schematic drawing of the DVSS. The DV is shunted into the IVC (**a**) and the HV (**b**)

**Fig. 17:**
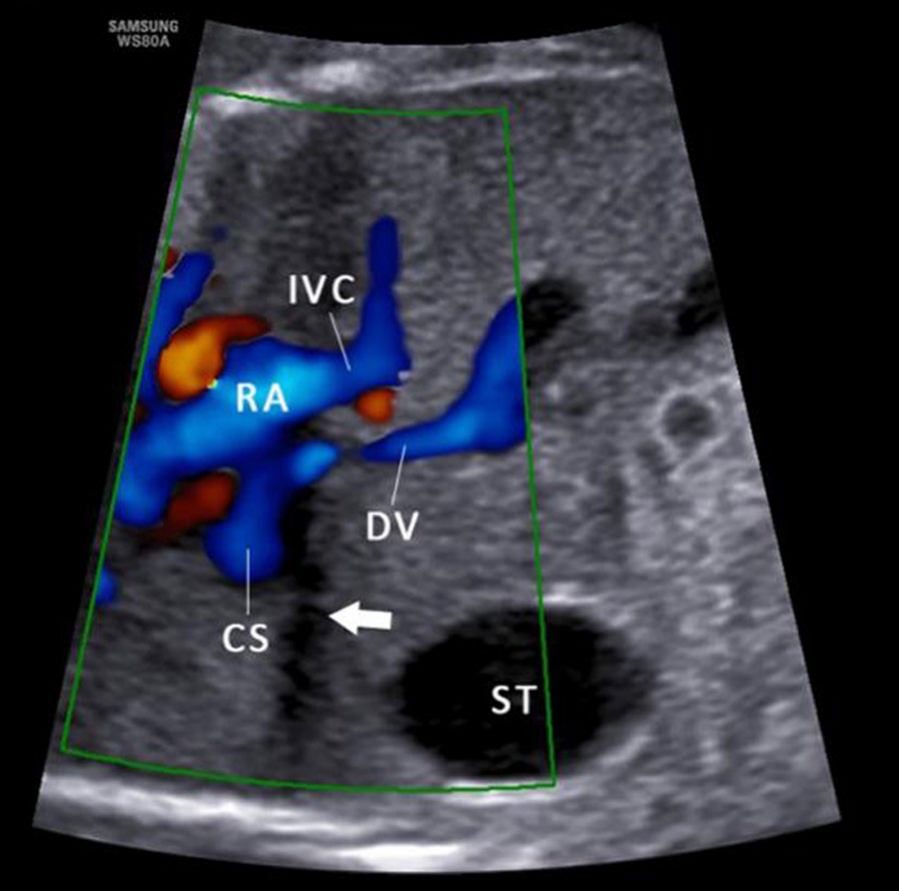
Color Doppler flow imaging of DVSS. The DV connected with the CS rather than the IVC

Ductus venosus-systemic shunts (DVSS) is referred as the slightly abnormal connection of the DV, which is shunted from its normal path to the hepatic fragment of the IVC, the abdominal IVC, the hepatic vein or the coronary sinus, with an intact the umbilical–portal-DV complex structure [9, 41] (Figs. 16, 17). This type is characterized by the presence of a normal IHPVS, in which the DVSS could differ from the USS. Therefore, some experts believe that the DVSS is a variation of normal anatomy. It has been reported that the fetuses with the DVSS are associated with a high incidence of chromosomal malformation and a low risk of other structural malformations. Genetic examination should be recommended for fetuses with DVSS detection to exclude chromosomal abnormalities. Fetuses with isolated DVSS have a good prognosis and normal liver function. Not all alive cases can be detected the shunt by targeted postnatal ultrasonography, and medical intervention is not necessary [41].

#### Congenital hepatoportal arteriovenous fistula

Congenital hepatoportal arteriovenous fistula (CHPAVF) is a kind of vascular malformation, of which the pathogenesis is the shunt between the hepatic artery and the portal venous system, leading a large amount of arterial blood drain into the PV, with the result of a rare cause of pediatric pre-sinusoidal portal hypertension and its complications. Prenatal ultrasound with Doppler might show single or multiple direct communications between the hepatic artery and the portal vein branches. Additional findings include hepatic artery enlargement, portal vein dilatation at the site of fistula, and abdominal aorta tapering beyond the celiac artery [45]. The main clinical symptoms of CHPAVF are shortness of breath, malaise, poor appetite and watery diarrhea [46]. CHPAVF can lead to high out-put heart failure with a mortality rate of 50–90% [46]. The prenatal diagnosis of CHPAVF enables better planning of postpartum management. Surgical resection, hepatic artery embolization and hepatic artery ligation have all been said to be important tools in the management of this condition [45–47].

#### Isolated absence or atresia of ductus venosus

**Fig. 18:**
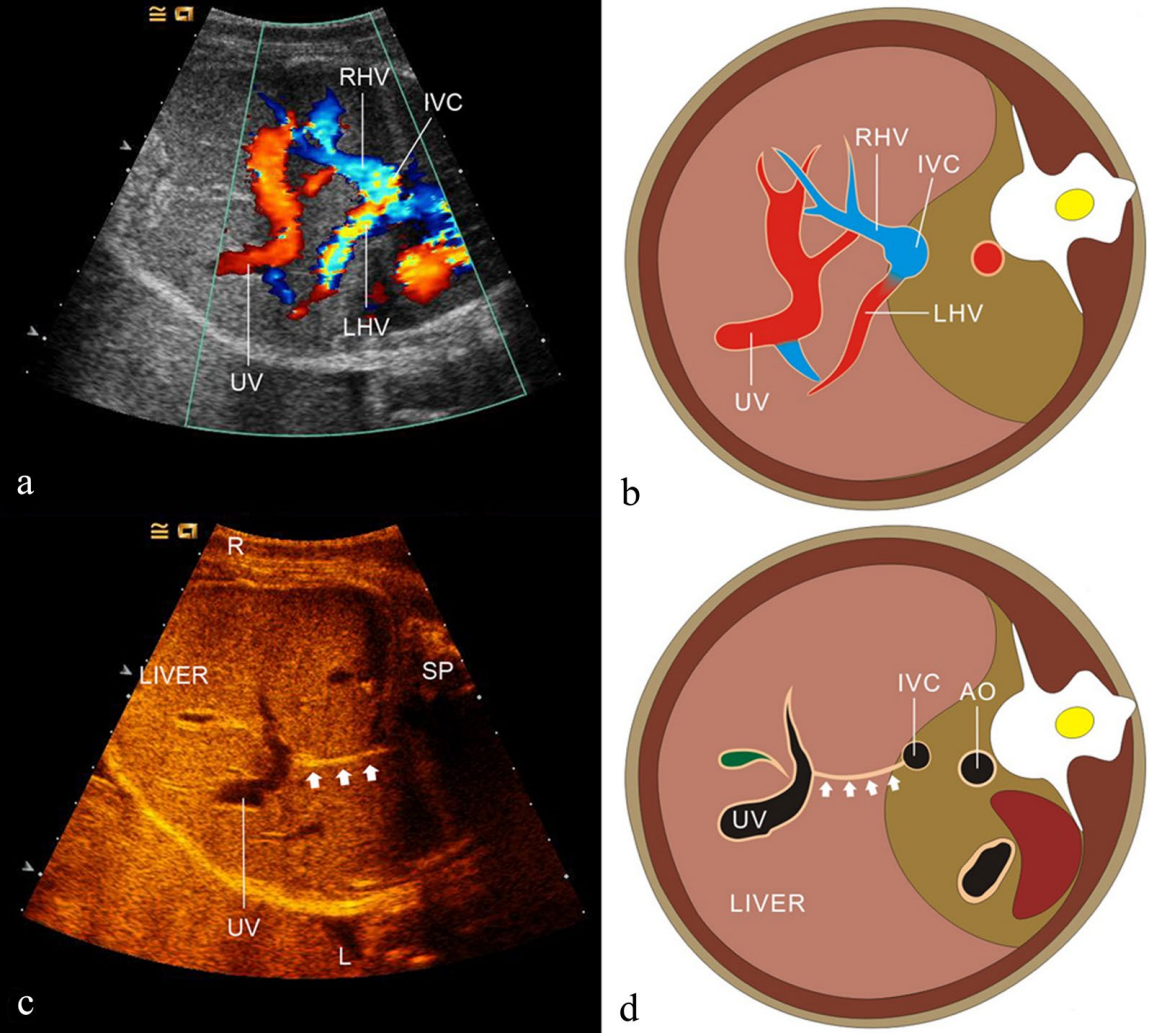
Sonographic images and schematic drawings illustrate isolated absence (**a**, **b**) and atresia (**c**, **d**) of DV

The DV plays an important role in fetal circulation because of diverting oxygenated blood from the placenta toward the right atrium and through the foramen ovale to the left heart and supporting the brain. When the DV is absent or atretic, the UV completely drains into the portal sinus connecting with the intrahepatic portal venous system [48]. Ultrasound picture demonstrates that the DV is absent or presents as a thin band connecting the LPV and the IVC (Fig. 18) and that CDFI examination could not demonstrate the blood flow signal. There is the research suggested that the isolated absence or atresia of DV had good prognosis in 67.2% cases and died in perinatal period as a result of fetal edema in 15.6% cases. However, the experts considered that this result might be exaggerated [48]. Prenatal ultrasonography detects the isolated absence or atresia of the DV, close surveillance of fetal hemodynamic changes is recommended. If the signs of fetal heart enlargement and fetal edema are detected, delivery must be performed as soon as possible.

#### Abnormal entry of the umbilical vein into the portal vein

**Fig. 19:**
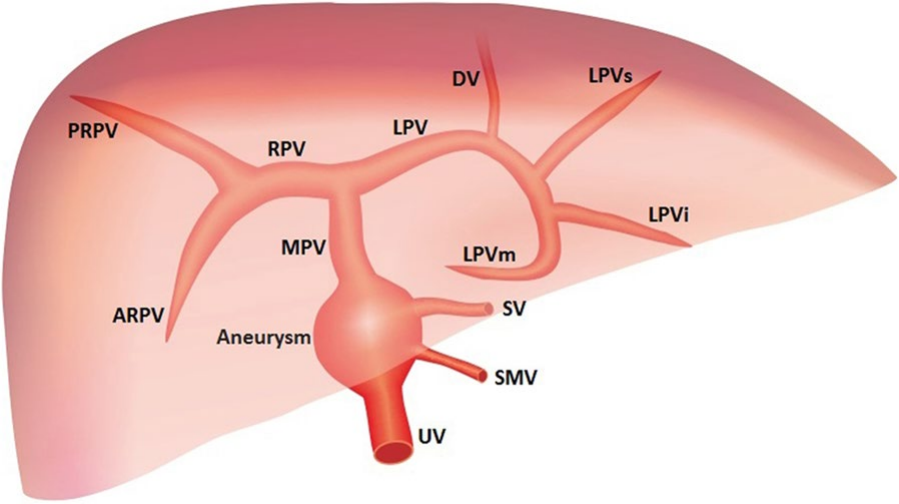
Scheme of the abnormal connection between the UV and the MPV. The confluence of the UV and the PV presents as the aneurismal dilatation

Abnormal connection of the UV and the PV is rare congenital vascular anomalies. Some experts concluded that an anastomosis between the left umbilical vein and the right vitelline vein at an early stage during embryogenesis was possible reason [49]. Most cases of this abnormality accompany with the aneurismal dilatation at the confluence of the UV and the PV. There is a variety of variations of influent blood vessel at the confluence, most are the SMV and the SV. The DV usually originates from the MPV [50, 51] (Fig. 19). Most specialists believe dilated extrahepatic vessel could be of vitelline, rather than umbilical system origin [49–51]. The anomaly is usually associated with an aneurysmal thrombosis, which can cause substantial postnatal morbidity, including portal hypertension, and even brain infarct. The case without thrombosis can be conservatively observed and followed up [51]. If a thrombosis is suspected, early surgical thrombectomy associated with vitelline vein resection is proposed. Surgery should take place as soon as the thrombus appears in order to avoid persistent portal thrombosis and its specific complications [49–53].

### Screening strategy

**Fig. 20:**
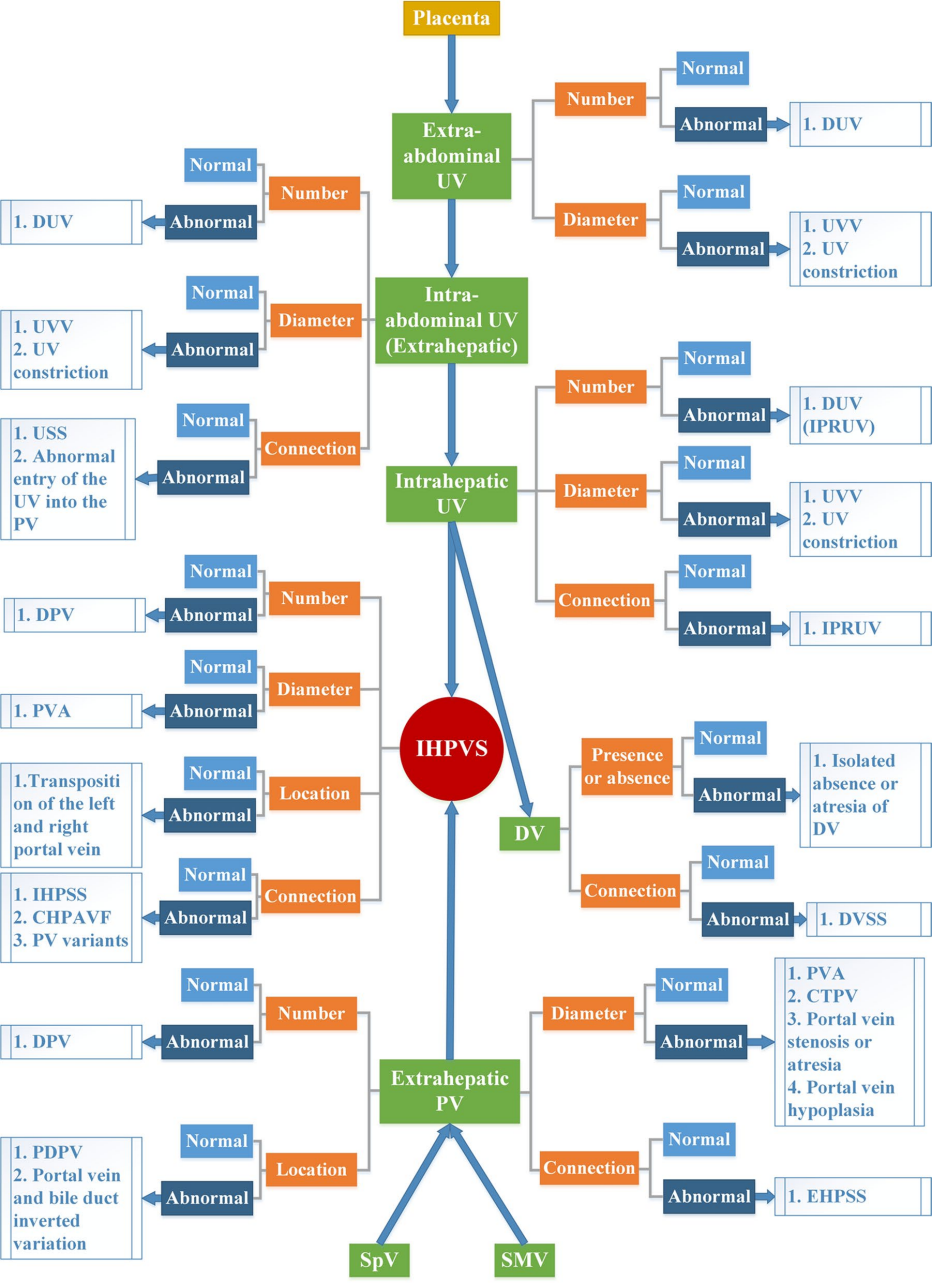
The detailed screening strategy framework

Two-dimensional ultrasound to detect the anatomical structures of UPVS is the essential first step in routine prenatal ultrasound examination. It mainly includes the following four main points: (1) number, diameter and connection site of the UV; (2) presence or absence and connection site of the DV; (3) number, diameter, connection and integrity of the IHPVS; (4) number, diameter, location and connection site of the extrahepatic PV. Color or HD-flow Doppler can routine use to explore the direction of blood flow. Pulse Doppler can be used to assess Doppler waveform and shunt size, especially in the case of CHPAVF. The detection of congenital abnormalities of UPVS increases significantly with a systematic examination and the use of Doppler ultrasound. The detailed screening strategy framework is shown in Fig. 20.

## Conclusion

The embryology of UPVS is complex, all types of developmental abnormalities and morphological variations are diverse, and there is overlap between different classifications. By summarizing previous classifications and relevant references, we propose a new classification of congenital abnormalities of UPVS. Some UPVS abnormalities have not been reported in relevant prenatal studies so far. In addition, it is too difficult to make a definitive prenatal ultrasonographic diagnosis of all abnormalities of UPVS. However, the new classification and screening strategy could give ultrasonographists a clue, when some anomalies are detected by prenatal ultrasound, the whole UPVS, the heart function and other anatomical structures should be performed detail examination. Once associated abnormalities are detected, prognosis needs to be reappraised, seeking to provide more information on prenatal counseling and subsequent management.

## Data Availability

The datasets used and/or analyzed during the current study are available from the corresponding author on reasonable request.

## Abbreviations

ARPV: Anterior right portal vein
AWV: Abdominal wall vein
CHPAVF: Congenital hepatoportal arteriovenous fistula
CIV: Common iliac vein
CS: Coronary sinus
CTPV: Cavernous transformation of portal vein
CVs: Cardinal veins
DPV: Duplication of the portal vein
DUV: Duplication of the umbilical vein
DV: Ductus venosus
DVSS: Ductus venosus–systemic shunts
EHPSS: Extrahepatic portal-systemic shunt
GB: Gallbladder
IHPSS: Intrahepatic portal-systemic shunt
HVs: Hepatic veins
IHPVS: Intrahepatic portal venous system
IMV: Inferior mesenteric vein
IUFD: Intrauterine fetal demise
IUGR: Intrauterine growth restriction
IVC: Inferior vena cava
LHV: Left hepatic vein
LPV: Left portal vein
LPVi: Inferior branch of the left portal vein
LPVm: Middle branch of the left portal vein
LPVs: Superior branch of the left portal vein
MHV: Middle hepatic vein
MPV: Main portal vein
PDPV: Preduodenal portal vein
PRPV: Posterior right portal vein
PRUV: Persistent right umbilical vein
PS: Portal sinus
PV: Portal vein
PVA: Portal vein aneurysm
RA: Right atrium
RHV: Right hepatic vein
RPV: Right portal vein
SMV: Superior mesenteric vein
SpV: Splenic vein
ST: Stomach
UPVS: Umbilical–portal venous system
USS: Umbilical–systemic shunts
UV: Umbilical vein
UVV: Umbilical vein varix
VVs: Vitelline veins
